# Acupuncture for chronic diarrhea in adults: Protocol for a systematic review

**DOI:** 10.1097/MD.0000000000005952

**Published:** 2017-01-27

**Authors:** Zongshi Qin, Bo Li, Jiani Wu, Jinhui Tian, Shang Xie, Zhi Mao, Jing Zhou, Tae-Hun Kim, Zhishun Liu

**Affiliations:** a Department of Acupuncture, Guang’anmen Hospital, China Academy of Chinese Medical Sciences; b Department of Gastroenterology, Beijing Hospital of Traditional Chinese Medicine Affiliated With Capital Medical University, Beijing Institute of Traditional Chinese Medicine, Beijing, China; c Korean Medicine Clinical Trial Center, Korean Medicine Hospital, Kyung Hee University, Seoul, Republic of Korea; d Evidence-Based Medicine Center, Lanzhou University, Lanzhou; e Department of Oral and Maxillofacial Surgery, Peking University School and Hospital of Stomatology, Beijing; f Department of Microsurgery, People's Liberation Army of China 205 Hospital, Jinzhou, China.

**Keywords:** acupuncture, chronic diarrhea, diarrhea-predominant irritable bowel syndrome, functional diarrhea, protocol, systematic review

## Abstract

**Background:**

As 2 major common types of chronic diarrhea, functional diarrhea (FD) and diarrhea-predominant irritable bowel syndrome (IBS-D) affect 1.54% to 1.72% of people in China. Acupuncture is commonly used in clinical practice for patients with chronic diarrhea. Here, we present a protocol of systematic review aimed at systematically review all the clinical evidence on the effectiveness of acupuncture for treating FD and IBS-D in adults.

**Methods:**

The review will be performed according to the Preferred Reporting Items for Systematic Reviews and Meta-Analyses Statement. We will search the following databases from their inception to January 2017: Cochrane Central Register of Controlled Trials, MEDLINE, EMBASE, China Biology Medicine disc, Wan-Fang Data, China National Knowledge Infrastructure, Citation Information by National Institute of Informatics, Oriental Medicine Advanced Searching Integrated System by Korea Institute of Oriental Medicine, and Japan Science and Technology Information Aggregator (J-stage). Clinical trial registrations will also be searched. Primary outcome measures are the change of bowel movements. The secondary outcomes include stool consistency, quality of life scales, other standardized rating scales, patient satisfaction, and acupuncture-related adverse effects assessment.

**Ethics and dissemination:**

This review does not require ethical approval and will be disseminated electronically or in print.

**PROSPERO registration number:**

CRD42015017574.

## Abstract 2

Supplemental Digital Content is available in the text

## 1 Introduction

Chronic diarrhea is a common complaint in worldwide, which is termed chronic when it lasts for <4 weeks.^[1,2]^ Owing to the epidemiological survey, chronic diarrhea has affected up to 26.9% of adults in the United States and increased the economic burden.^[3]^ The 2 major types of chronic diarrhea are functional diarrhea (FD) and diarrhea-predominant irritable bowel syndrome (IBS-D).^[4]^ The predominant symptoms of these 2 illness are differentiable that IBS-D is distinguished by the recurrent abdominal pain/discomfort during diarrhea with a relief after the defecation, or a change of the bowel habits; and FD is characterized by the frequent presence of loose or watery stools (<75%) with or without abdominal pain.^[4,5]^ Although these 2 illnesses are differentiable through Rome Criteria, IBS-D and FD still have considerable overlapped symptoms of chronic diarrhea such as loose or watery stools, increased stool frequency, and bloating.^[6]^ Based on a cross-sectional survey conducted in 16,078 respondents, the prevalence of FD and IBS-D is 1.72% and 1.54% in China, respectively.^[7]^ To date, the underlying pathophysiology is still unclear, and the development of FD and IBS-D may involve rapid intestinal transit, emotions, or specific nutrient intolerances.^[8]^

Recently, the treatments of chronic diarrhea are focused on symptom management, which can be classified as dietary and lifestyle modification, psychological therapies, pharmacologic therapies, and alternative modalities.^[9]^ Medications like anticholinergics, antimotility drugs, and antidiarrheal agents may be beneficial for FD and IBS-D symptoms.^[10–12]^ However, the adverse effects including sleepiness, dizziness, nausea, vomiting, constipation, dependency, tolerance, and respiratory depression,^[13,14]^ which might cause discontinuation of the medication.^[15,16]^

Acupuncture is a nondrug technique among various interventions in complementary and alternative medicine. Most commonly, acupuncture is accomplished by manual manipulation or electrical stimulation via thin needles inserted in acupuncture points.^[17]^ Acupoints are located on meridians and the theory behind acupuncture was outlined in Inner Canon of Huangdi (an ancient medical textbook).^[18]^ Studies suggested that acupuncture may be effective for gastrointestinal diseases,^[19–21]^ and a systematic review reported that the quality of life in patients with gastrointestinal diseases was improved significantly, independently through acupuncture method.^[22]^ Some animal trials manifested that acupuncture could increase or decrease the intestinal motility.^[23,24]^ The mechanism of acupuncture on gastrointestinal motility seemed to be mediated via neural and humoral pathways, and some researchers found that acupuncture involved the central nervous system, autonomic nervous system, and enteric nervous system.^[20,25–27]^ In this review, we aim to perform a systematic review to assess and appraise all the clinical evidence on the effectiveness and safety of acupuncture for treating FD and IBS-D in adults.

## 2 Methods

This systematic review protocol has been registered on PROSPERO under the number CRD42015017574, and has been developed following the Preferred Reporting Items for Systematic Reviews and Meta-Analyses protocols (PRISMA-P) statement guidelines.^[28]^ (see file 1, Supplemental Content, which represents the PRISMA-P checklist)

### 2.1 Criteria for including studies in this review

#### 2.1.1 Types of studies

We will include all original studies published in English, Chinese, Korean, and Japanese that reported the effectiveness and/or safety on acupuncture for FD or IBS-D, only randomized controlled trials (RCTs) will be included in data syntheses. To RCTs, it should report adequate randomization methods, eligible diagnosis, eligible outcome measurement, and statistical methods description. We will also search the database to include nonrandomized study, which can be divided into nonrandomized intervention study and observational study (indicating quasi-RCT, clinical controlled trials, and cohort studies). For all nonrandomized study, we will only extract the data from acupuncture group for effectiveness and safety assessment without data syntheses. The details will be described in the “Data syntheses” section and “Assessment and quality of included studies” section. Trials without established standard international diagnoses and/or outcome measures will be excluded.^[29,30]^

#### 2.1.2 Types of participants

Trials must include adult participants (more than 18 years old) with chronic diarrhea. In this review, we mainly focus on participants who were diagnosed as FD or IBS-D.

For participants with FD: with loose (mushy) or watery stools, increased stool frequency without pain occurring in at least 75% of stools lasting more than 3 months.

For participants with IBS-D: recurrent abdominal pain or discomfort at least 3 days per month in the last 3 months associated with improvement with defecation, onset associated with a change in frequency or appearance (evaluated with Bristol scale type 5 to 7, if available) of stool.^[29]^ In addition to this, loose stool needed to be >25% and hard stool to be <25% of the time for this participants.^[30]^

#### 2.1.3 Types of interventions

Any type of penetrating acupuncture will be included, such as acupuncture, electro-acupuncture, auricular acupuncture, abdominal acupuncture, and warm acupuncture. We will exclude nonpenetrating acupuncture such as laser acupuncture, acupressure, and transcutaneous or percutaneous electrical nerve stimulation. Control interventions will include no treatment, sham acupuncture/placebo acupuncture (e.g., puncture the same acupuncture point as treatment group without needle insertion or puncture the areas not corresponding to any real acupuncture points), and conventional drug therapies (e.g., intestinal transit inhibitors, intraluminal agents, proabsorptive agents, antisecretory drugs). Either sham acupuncture or placebo acupuncture will be seen as the sham controlled group instead of a sort of acupuncture, we will also exclude the trials that compare 2 different types of acupuncture (such as acupuncture vs. electro-acupuncture).

#### 2.1.4 Types of outcomes

We will include trials that report at least one of the following outcomes.

The primary outcome is the change from baseline in weekly bowel movements at the end of the treatment or follow-up.

The secondary outcomes include

(1) The score change from baseline in stool consistency assessed through Bristol Stool Form Scale (BSFS, score ranged from 1 to 7 for stool types 1 to 7, respectively).^[31]^ According to BSFS, stool consistency is classified into 7 types, in which, type 5 to 7 denote the stool type of diarrhea, and type 4 denotes a normal stool consistency.^[32]^
(2) Score change from baseline in quality of life measurement.
(3) Other standardized rating scales (e.g., IBS Adequate Relief question, IBS Symptom Severity Scale).
(4) Patient satisfaction at the end of treatment.
(5) Acupuncture-related adverse effects assessment such as broken or left needle; hypodermal bleeding, hematoncus, or infection around the site of needling; unbearable pricking, faint, nausea, or dizziness during the needling; injury to organs during or after acupuncture; other discomfort such as fatigue, or sleeplessness after the acupuncture.

For the studies from China used “Criteria of diagnosis and therapeutic effect of diseases and syndromes in traditional Chinese medicine” to report outcomes based on a fuzzy assessment such as “cured,” “improved,” and “failed” will be excluded.

### 2.2 Search methods for identification of studies

#### 2.2.1 Electronic searches

The search strategy of Chinese, English, and Japanese will be developed by ZQ, JT, and SX, the search strategy of Korean will be developed by T-HK. The following databases will be searched from their inception to January 2017: the Cochrane Central Register of Controlled Trials, MEDLINE, EMBASE, Chinese Biology Medicine disc, Wan-Fang Data, China National Knowledge Infrastructure, Citation Information by National Institute of Informatics, Oriental Medicine Advanced Searching Integrated System by Korea Institute of Oriental Medicine, and Japanese database Science and Technology Information Aggregator (J-stage). In addition, we will also search other electronic sources of trial registries, including Clinicaltrials.gov (http://www.clinicaltrials.gov) and International Clinical Trials Registry Platform (http://www.who.int/trialsearch/Default.aspx). Besides, before this review will be finished, we will search each database and registration plat once again to ensure the latest study could be included (see file 2, Supplemental Content, which represents the search strategy for PubMed database, similar search strategies will be applied for the other databases, http://links.lww.com/MD/B534).

### 2.3 Data collection and analysis

#### 2.3.1 Selection of studies

**Figure 1:**
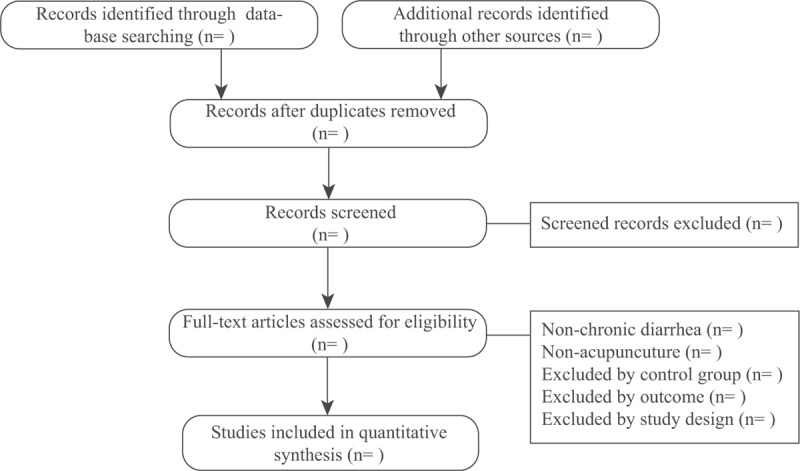
PRISMA flow chart.

For different language database, 2 review authors (ZQ and BL for Japanese database; JW and JZ for English database, ZM and XS for Chinese database, and T-HK and JT for Korea database) will screen the title and abstracts of the articles independently to confirm that the trials included are eligible. If necessary, the full text will be scanned. We will use Endnote X7 (Thomson Reuters, New York, NY) software to manage the trials that have been searched and remove duplicates. Any disagreement with the selection will be discussed and judged by an arbiter (ZL). The details of the selection process are shown in Fig. 1.

#### 2.3.2 Data extraction

For different language database, 2 review authors (same as “Selection of studies” section) will independently extract the data. Before beginning extraction, a consistency assessment between these 2 authors will be performed through a pilot test, in which each of them will evaluate 2 trials, respectively. After a common consensus is reached, we will use a predefined extraction form to collect data from included trials (see file 3, Supplemental Content, which represents the electro-sheet for data extraction, http://links.lww.com/MD/B535), including general information (name and year of publication, date of extraction, title of study, and author's publication details), study characteristics, eligibility criteria, interventions, outcome measurements, duration, adverse events, results, and type of needle used. Any disagreements will be discussed and judged by an arbiter (ZL or T-HK).

#### 2.3.3 Assessment and quality of included studies

The Cochrane Collaboration tool for assessing the risk of bias will be used to facilitate the assessment of risk of bias for the included RCTs.^[33]^ For different language database, 2 review authors (same as “Selection of studies” section) will independently evaluate the quality. The result of the consistency assessment will be presented with Kappa statistics. Kappa value <0.75 will be considered that the 2 authors are consistent. The evaluation of the trials’ quality will cover 7 aspects: random sequence generation, allocation concealment, blinding of participants and personnel, blinding of outcome assessment, incomplete outcome data, selective reporting, and other bias. Additionally, we will use the GRADEpro software of Cochrane Systematic Reviews to create a Summary of Findings Tables, and the Summary of Findings Tables will be integrated in the RevMan. For nonrandomized study, we will use Newcastle–Ottawa Quality Assessment Scale (NOS) for assessing the observational study (including cohort studies), or methodological index for nonrandomized studies (MINORS) for assessing the nonrandomized interventional study.^[34,35]^ Any disagreements will be discussed and judged by an arbiter (ZL or T-HK).

#### 2.3.4 Unit of analysis issues

If there are cross-over trials, we will only analyze the data from the first period. When there are more than 2 arms in 1 trial, we will choose data of the arm that meet our inclusion criteria for data synthesis. If there are multiple observations, we will extract the short-term effect (at the end of treatment) and long-term effect (at the end of follow-up) for analyzing.

#### 2.3.5 Dealing with missing data

Review authors who are responsible for different language databases will make every effort to obtain the missing information from the trials including sending emails or calling the authors to ask for the missing data. For trials that provide data of baseline and end-point without change value, we will estimate the approximate value of changes using the methods recommended in the Cochrane Handbook.^[36]^ When studies reported standard errors, *t*-statistics, or *P* values without standard deviations (SDs), we will transform them into SDs. For the trials meet our inclusion criteria without available date, we will only provide the characteristics in the supplementary without data extraction and synthesis.

#### 2.3.6 Assessment of heterogeneity

We will use Higgins I^2^ statistic to test clinical heterogeneity. Variability factors included in the trials will be taken into consideration (e.g., type of intervention, duration of intervention). I^2^ ≥ 50% will be considered indicative of substantial heterogeneity among the trials,^[36]^ and we will explore the source of the heterogeneity from the design of trials and characteristics in the included trials through conducting sensitive analysis or subgroup analysis.

#### 2.3.7 Assessment of reporting bias

We will use Funnel plot to assess the reporting bias if a sufficient count of the included RCTs is available (10 or more trials are included in a meta-analysis).

#### 2.3.8 Data synthesis

We will use the RevMan V.5.3.4 to perform meta-analysis for RCTs included if studies are sufficiently homogeneous in terms of design and comparator, we will explore the homogeneous from following aspects: The clinical homogeneous, which will be judged by professional and experienced assessor and described in the characteristic summary table; the second respect is methodological homogeneous, which will be assessed by the Cochrane Q-test and quantified with I^2^ value. The dichotomous data will be determined by using relative risk with 95% confidence interval (CI), and continuous outcomes will be analyzed using standard mean difference with 95% CI. For each outcome, we will initially assume that each meta-analysis comparing acupuncture group and control group has its own heterogeneity variance parameter τ^2^ using fixed-effects model. When the heterogeneity is significant, we will use a random-effects model. When we meet the situation that quantitative synthesis is not appropriate such as insufficient RCTs or significant heterogeneity that cannot be identified, we will provide systematic narrative synthesis to describe the characteristics and findings of the included trials. For nonrandomized study, the data will not be combined, as it may result in inestimable heterogeneity due to clinical and methodological differences. However, we will generate visual results of forest plots to present the data from different studies to show the direction and magnitude of effects.

#### 2.3.9 Other analysis

Sensitivity analysis will be performed to assess whether there is significant heterogeneity, we will exclude trials rated as low or unclear risk of bias in the allocation concealment domain and then reassess the before and after outcome from the data synthesis to observe the heterogeneity in the synthesis of remained trials. Furthermore, we will use subgroup analysis to explore the sources of heterogeneity in following fields: the data from the participants with FD and those with IBS-D will be analyzed separately after data combination; duration of intervention will be considered, we will classify the trials into 2 subgroups based on the treatment duration. Additionally, if the number of included trials is available (10 or more trials are included in a meta-analysis), meta-regression analysis will be conducted using Stata software version 12.0 (Stata Corporation, College Station, TX) to explore sources of heterogeneity.

## 3 Discussion

The cause of FD and IBS-D is still unclear and there are few specific treatment aside from symptomatic management to prevent electrolyte and water loss. Numerous pharmacologic treatments can be used in these patients; however, comparative studies establishing a hierarchy of cost–benefit and a sequence of use of such agents are still lacking.^[8]^ In addition to pharmacological treatment, there are some complementary and alternative approaches that may be effective to gastrointestinal disease.^[37,38]^ Studies have indicated that acupuncture has effects on some digestive system diseases.^[13,39]^

One systematic review reported that acupuncture might be more effective than drugs on treating FD from total effective rate, antidiarrheal time, and symptom total scores aspects.^[40]^ However, the measurement of effective rate used in this study is assessment of global improvement instead of subjective outcomes such as stool frequency. Another systematic review of IBS reported that acupuncture is not superior to sham acupuncture on IBS symptom severity or IBS-related QoL.^[41]^ But this review did not separate IBS-D from constipation predominant IBS, and did not provide a subgroup analysis of IBS-D as well, which might not reveal the effectiveness of acupuncture for IBS-D adequately. It is hard to distinguish FD and IBS-D absolutely in the clinical practice for the considerable overlapped symptoms between them. Acupuncture might manage the symptoms of diarrhea through regulating the bowel motility, and the mechanism of acupuncture treatment for FD and IBS-D might be similar.^[21]^ Owing to the study focus on both FD and IBS-D is rare, the conclusion that if acupuncture could be an optional treatment for FD and IBS-D is still unclear.

Therefore, we aim to conduct this systematic review to assess the effectiveness and safety of acupuncture in the treatment of FD and IBS-D. Besides, considering the insufficient amount of potential RCTs, we will also include nonrandomized study that meet our criteria set in advance, in order to provide a visual representation of results to show the direction and magnitude of effects; thus, the results could be more enriched. We will generate an assessment table using NOS and MINORS to evaluate the quality of included nonrandomized study. We hope that this systematic review can provide a convincing conclusion and summarize the evidence on the effectiveness and safety of acupuncture on this disorders for clinicians, patients, and health policy conductors.

## Supplementary Material

Supplemental Digital Content
